# The relationship between depression symptoms and academic performance among first-year undergraduate students at a South African university: a cross-sectional study

**DOI:** 10.1186/s12889-022-14517-7

**Published:** 2022-11-11

**Authors:** F Wagner, RG Wagner, U Kolanisi, LP Makuapane, M Masango, FX Gómez-Olivé

**Affiliations:** 1 Analytics and Institutional Research Unit (AIRU), University of the Witwatersrand, Johannesburg, South Africa; 2 MRC/Wits Rural Public Health and Health Transitions Research Unit (Agincourt), School of Public Health, Faculty of Health Sciences, University of the Witwatersrand, Johannesburg, South Africa; 3 Faculty of Science and Agriculture, Department of Consumer Sciences, University of Zululand, 3886 KwaDlangezwa, South Africa; 4 Department of Institutional Planning (DIP), University of Pretoria, Pretoria, South Africa

**Keywords:** College students, Depression, Higher education institution, Mental health, Progression

## Abstract

**Background:**

South African universities face a challenge of low throughput rates, with most students failing to complete their studies within the minimum regulatory time. Literature has begun to investigate the contribution of well-being, including mental health, with depression among students being one of the most common mental disorders explored. However, locally relevant research exploring associations between depression and academic performance has been limited. This research hypothesizes that the presence of depression symptoms, when controlling for key socio-demographic factors, has an adverse impact on student academic outcomes and contributes to the delay in the academic progression of students.

**Methods:**

The study used a cross-sectional design. Data were collected in 2019 from first-time, first-year undergraduate students using a self-administered online questionnaire. In total, 1,642 students completed the survey. The Patient Health Questionnaire-9 (PHQ-9) was used to screen for depression symptoms. Data on students’ academic performance were obtained from institutional records. Bivariate and multivariate regression analyses were used to examine associations between depression symptoms and academic performance.

**Results:**

Most participants (76%) successfully progressed (meeting the requirements to proceed to the second year of university study). Of the participants, 10% displayed symptoms of severe depression. The likelihood of progression delay (not meeting the academic requirements to proceed to the second year of university study) increased with the severity of depression symptoms. Moderate depression symptoms nearly doubled the adjusted odds of progression delay (aOR = 1.98, 95% CI: 1.30-3.00, *p* = 0.001). The likelihood of progression delay was nearly tripled by moderate severe depression symptoms (aOR = 2.70, 95% CI:1.70–4.36, *p* < 0.001) and severe depression symptoms (aOR = 2.59, 95% CI:1.54–4.36, *p* < 0.001). The model controlled for field of study, financial aid support as well as sex and race.

**Conclusion:**

Higher levels of depression symptoms among first-year university students are associated with a greater likelihood of progression delay and may contribute to the low throughput rates currently seen in South African universities. It is important for students, universities and government departments to recognize student mental wellness needs and how these can be met.

**Supplementary Information:**

The online version contains supplementary material available at 10.1186/s12889-022-14517-7.

## Background

Mental illness is a public health priority, affecting as much as 47% of the population at some point in their lifetime [1]. Literature has identified university students as a group that is particularly vulnerable to mental illness [2–6]. A review on depression among university students reported that depression prevalence ranged between 6 and 54% among university students [6]. Depression is a disorder that can affect one’s overall functioning. Symptoms of depression can often include a lack of a positive outlook, high levels of anxiety, irregular sleeping patterns and reduced concentration [4, 7]. The high prevalence of depression among university students is concerning and justifies a need to understand how depression and academic success in this population may be associated.

There is no agreed-upon definition for academic success or failure, but there is consensus that, traditionally, student academic success is represented by student retention, progression and improved throughput; while academic failure can be described as the lack of retention, progression or throughput [8–10]. In the South African context, universities are faced with low throughput rates, meaning that only a small percentage of students obtain their qualifications within the minimum stipulated times. This is largely due to progression delay, a consequence of students not meeting the academic requirements to progress from one academic year to the next [9]. These delays in progression are particularly significant in the South African context, where more than half of the population lives in poverty and most young people are unemployed [11, 12]. In this context, unemployment is lowest among those with tertiary qualifications [12] and thus delays in acquiring qualifications can be devasting for students coming from poor homes, who are often expected to support their families financially upon graduation. It is therefore imperative that university student success is prioritised.

Several studies have explored factors and determinants of throughput and student success [13–16]. These studies found that the determinants of student success are complex, identifying high school academic achievement [13, 17], family background [15] and the students’ ability to integrate into the different aspects of university life, including social communities and teaching and learning [14] as key determinants of academic success.

In addition to these traditional determinants, research has begun to explore the contribution of well-being, including mental health, as a potential contributor to student academic performance. This emerging research suggests that university students suffering from common mental disorders, especially anxiety and depression, are likely to perform poorly when compared to students without mental disorders [2, 3, 18]. A study among university students in the United States of America (USA), found that depression was not only linked to a lower grade point average (GPA), but also an increased likelihood of attrition [2]. Findings from a longitudinal cohort study in the United Arab Emirates (UAE) found that higher levels of depression predicted lower GPA scores both at baseline and follow-up [19]. Work carried out in South Africa found that students with major depressive disorder as well as those with attention deficit hyperactivity disorder (ADHD) had a higher probability of academic failure [3]. Research conducted in Australian universities found students often attributed their academic failure to poor mental health, including conditions such as anxiety and depression [20]. Evidence from students in Nigeria found depression to be inversely linked to perceived poor academic performance [21].

South African literature on depression and associations between depression and academic failure among university students has started to emerge [3, 22, 23]. However, findings from these studies have been based predominantly on White study participants, making it difficult to generalize these findings to more heterogenous student populations since White students are in minority in the South African higher education sector. Given this, the current research aims to close this knowledge gap while considering other important factors, such as financial aid and field of study, which may impact on progression delay. We hypothesize that the presence of depression symptoms has adverse effects on student academic outcomes and contributes to progression delay in a diverse South African university student population. The main aim of the current study was therefore to investigate the extent to which depression symptoms, when controlling for key demographic and socio-economic factors, predicted student progression delay.

## Methods

The current study took place in a large research-intensive South African university. In 2019, the headcount student enrolment was around 41,000, with international students making up 9% of the student population. Female students made up 55% of the student population, and the majority of South African students attending the university were Black African (61%). The student population is culturally diverse, with the university having three official languages, English, IsiZulu and Sesotho.

### Sample

The current research targeted the entire cohort of 2019 first-time, first-year undergraduate students (n = 5,912). The inclusion criteria to participate in this research, which was used to extract the sample, was as follows: being 18 years of age or older; being a first-time, first-year undergraduate student; studying full-time; completion of the Biographic Questionnaire [16], which is a baseline survey at intake; pursuing either a professional bachelor’s degree (a programme that is generally four years or longer) or a general bachelor’s degree (generally a three-year programme); and being assigned progress codes at the end of the 2019 academic year. All students provided informed consent prior to participating in the study. Students not meeting the inclusion criteria were excluded.

All students meeting the inclusion criteria were invited to participate in the study (n = 5,195). Students could only complete the survey once they had given informed consent. A total of 1,648 participants (32%) completed the survey. Six participants were not assigned progress codes at the end of the 2019 academic year, the possible reasons for this include students deregistering or awaiting the outcome of appeals. The six records were removed from the analysis, leaving a total analysis sample of 1,642 participants.

A comparison between study participants and those who did not participate in the study (non-participants) (Table 1) shows that non-participants were significantly older (X^2^ (2, N = 5 195) = 14.95, *p* = 0.001), more likely to be male ((45% vs. 37%); X^2^ (1, N = 5 195) = 32.31, *p* < 0.001), less likely to be Black African ((64% vs. 72%); X^2^ (5, N = 5 195) = 28.87, *p* < 0.001), and were significantly more likely not to be receiving financial aid ((59% vs. 51%); X^2^ (1, N = 5 195) = 27.17, *p* < 0.001). The participant group was significantly more likely to be from high school quintiles 1–4, and significantly more likely to be first-generation students ((47% vs. 53%); X^2^ (1, N = 5 195) = 15.91, *p* < 0.001). There were no differences in disability status or field of study.

**Table 1:** Descriptive summary of characteristics by participants and non-participants

Variable	Non-Participants (n = 3 553)	Participants (n = 1 642)	*p-value*
**Sex (%)**			< 0.001
Female	1 952 (55%)	1 040 (63%)	
Male	1 600 (45%)	602 (37%)	
Sex not recorded	1 (0%)	0 (0%)	
**Race (%)**			< 0.001
Black African	2 288 (64%)	1 178 (72%)	
Chinese	18 (0%)	7 (0%)	
Coloured	150 (4%)	50 (3%)	
Indian	499 (14%)	198 (12%)	
White	565 (16%)	196 (12%)	
Unknown	33 (1%)	13 (1%)	
**Age (%)**			0.001
18	835 (24%)	462 (28%)	
19	2 025 (57%)	905 (55%)	
≥20	693 (19%)	275 (17%)	
**Reporting as disabled or having special needs (self- reported) (%)**			0.873
Yes	101 (3%)	45 (3%)	
No	3 452 (97%)	1 597 (97%)	
**High school quintile** **(%)**			0.007
1	205 (6%)	113 (7%)	
2	316 (9%)	172 (10%)	
3	517 (15%)	269 (16%)	
4	403 (11%)	200 (12%)	
5	1 215 (34%)	534 (33%)	
Other	897 (25%)	354 (22%)	
**Generation status (%)**			< 0.001
1st generation student	1 476 (42%)	779 (47%)	
2nd generation or more	2 077 (58%)	863 (53%)	
**Field of study (%)**			0.147
Commerce, Law and Management	556 (16%)	243 (15%)	
Engineering	804 (23%)	334 (20%)	
Health Sciences	509 (14%)	252 (15%)	
Humanities	956 (27%)	485 (30%)	
Science	728 (20%)	328 (20%)	
**NSFAS financial aid support recipient (%)**			< 0.001
Yes	1 455 (41%)	799 (49%)	
No	2 098 (59%)	843 (51%)	

In the analysis sample (Table 1) most study participants were female (63%), Black African (72%), between the ages of 18 and 39 (median 19 years), non-first-generation students (53%), attended high school quintile 5 (33%) and reported having no special needs (98%).

### Instruments

#### Depression

the Patient Health Questionnaire-9 (PHQ-9) was used to screen for depression symptoms using a two-week recall period [24]. The PHQ-9 has been validated and determined to give accurate accounts of the prevalence of depression symptoms [25]. Responses to each of the questionnaire items were rated on a four-point Likert scale, ranging from 0 (not at all) to 3 (nearly every day) [25]. Participants’ responses were summed and designated to one of five categories for the PHQ-9 (that is a PHQ-9 score of 0–4 denoting minimal depression symptoms; 5–9 denoting mild depression symptoms, 10–14 denoting moderate depression symptoms, 15–19 denoting moderate-severe depression symptoms, and 20–27 denoting severe depression symptoms), these categories have been used in other studies [26].

Covariates potentially influencing academic performance associations were identified in literature and included in the models. These covariates included: race (coded as Black African, Chinese, Coloured, Indian, White or Unknown), sex (coded as male or female), first-generation status (coded as 1st generation student for those first in their family to go to university), and 2nd generation or more (coded for those with family members who had attended university). A self-reported account of disability status was also included (coded as ‘yes’ for participants with self-reported disabilities and/or special needs, or ‘no’ for participants with no disabilities and/or special needs). Other covariates included field of study (coded as Commerce, Law and Management, Engineering, Health Sciences, Humanities, or Science), and financial aid from the National Student Financial Aid Scheme (NSFAS) (coded as ‘yes’, for those who were funded, or ‘no’ for those who did not receive funding).

#### Anxiety

Anxiety symptoms were measured using the GAD-7 questionnaire [27] which is a seven-item tool used to screen for anxiety symptoms. Like the PHQ-9, the GAD-7 uses a two-week recall period. Responses to each of the questionnaire items were rated on a four-point Likert scale, ranging from 0 (not at all) to 3 (nearly every day). Participants’ responses were summed and designated to one of four categories for the GAD-7 (with severity scores as follows: a score of 0–4 denoting minimal anxiety, 5–9 denoting mild anxiety, 10–14 denoting moderate anxiety and 15–21 denoting severe anxiety), these categories have been used in other studies [26, 27].

Students’ main source of general support was also included as a variable from the question: *While you are at university, who will be providing you with general support?* Participants had the following response options: Both parents, Single parents, Grandparent(s) or, Guardian(s), Other family and/or friends(s), Spouse/ partner, No support. General support, in this instance, means the support given to students in general, without any particular sub-divisions.

High school socio-economic quintile was also included. South African public schools are allocated quintile categories to reflect the socio-economic status of communities surrounding the schools. Quintile 1 represents the poorest communities and quintile 5 the wealthiest [17]. In addition to the high school quintiles 1–5, was the category ‘other’. The ‘other’ category included participants from non-public high schools (private and international high schools).

#### Academic performance

progress codes assigned to each student at the end of the academic year were dichotomized as (i) those meeting the requirements to proceed to the second academic year of study (successful progression) and (ii) those who did not meet the academic requirements to proceed to the second year of study (progression delay). This definition has been previously used to define academic success (here defined as ‘successful progression’) and academic failure (here defined as ‘progression delay’) in similar work in South Africa [3, 28].

### Procedure

Following ethics approval, as well as written permission from the university registrar, all first-time, first-year undergraduate student email addresses were extracted from the university database using the inclusion criteria stated above. Students were then invited to participate in the study via an email with a unique link to the survey. Students could only complete the survey after consenting (by clicking that they consented to take part in the study). Data collection, which took place over six weeks between July and August 2019, was in the form of a self-administered online questionnaire, which was hosted on the Research Electronic Data Capture (REDCap) web application [29]. Academic performance data (for the 2019 academic year) were requested from the university for students who completed the survey, this performance data was then linked to survey data.

### Data analysis

Data were cleaned and analyzed using STATA (version 14; College Station, Texas, USA). Frequency and descriptive analyses were performed for demographic and mental health variables. Categorical variables were reported using percentages and continuous variables were reported using the median and interquartile ranges (IQR). The Mann-Whitney U test was used to compare continuous variables, while the chi-square test was used to compare categorical variables with student progression. Variables included in the logistic regression model, which used adjusted odds ratios (aOR) as a test statistic, were selected using a forwards and backwards stepwise regression, with a cut-off of p$\le$0.20 used for inclusion in the model. Significance was defined at an p-value$<$0.05 level in all analyses.

## Results

As shown in Table 2, a total of 76% of students progressed successfully, while 24% experienced progression delay. A higher proportion of male students (31%) experienced progression delay, compared to female students (21%). Black African students and students from quintile 1 high schools had the highest proportion of progression delay at 27% and 33% in their respective groupings. In terms of field of study, students registered for programmes in the humanities had the lowest proportion of progression delay at 11%. There were significant differences between progression delay in the distribution by sex (X^2^ (1, N = 1 642) = 20.07, *p* < 0.001), race (X^2^ (5, N = 1 642) = 23.06, *p* < 0.001), high school quintile (X^2^ (5, N = 1 642) = 34.89, *p* < 0.001) and field of study (X^2^ (4, N = 1 642) = 228.20, *p* < 0.001).

**Table 2:** Descriptive summary of characteristics of the sample

Variable	Successful Progression (n = 1240)	Progression Delay (n = 402)	*p-value*
**Academic performance (%)**	1 240 (76%)	402 (24%)	
**Sex (%)**			< 0.001
Female	823 (79%)	217 (21%)	
Male	417 (69%)	185 (31%)	
**Race (%)**			< 0.001
Black African	856 (72%)	322 (27%)	
Chinese	7 (100%)	0 (0%)	
Coloured	40 (80%)	10 (20%)	
Indian	156 (79%)	42 (21%)	
White	170 (87%)	26 (13%)	
Unknown	11 (85%)	2 (15%)	
**Age in years (IQR)**	19 (18–19)	19 (18–19)	0.355
**Reporting as disabled or having special needs (self- reported) (%)**			0.486
Yes	32 (71%)	13 (29%)	
No	1 208 (76%)	389 (24%)	
**High School Quintile** **(%)**			< 0.001
1	76 (67%)	37 (33%)	
2	118 (69%)	54 (31%)	
3	182 (68%)	87 (32%)	
4	143 (72%)	57 (29%)	
5	432 (81%)	102 (19%)	
Other	289 (82%)	65 (18%)	
**Generation status (%)**			0.061
1st generation student	572 (73%)	207 (27%)	
2nd generation or more	688 (77%)	195 (23%)	
**Field of study (%)**			< 0.001
Commerce, Law and Management	212 (87%)	31 (13%)	
Engineering	164 (49%)	170 (51%)	
Health Sciences	216 (86%)	36 (14%)	
Humanities	434 (89%)	51 (11%)	
Science	214 (65%)	114 (35%)	
**NSFAS Financial aid support recipient (%)**			0.784
Yes	601 (75%)	198 (25%)	
No	639 (75%)	204 (24%)	

Age is described using median, Interquartile ranges and the Mann Whitney U test; the chi-square test was used to compare categorical variables to progression

In terms of mental health, the prevalence of severe anxiety symptoms was found to be 18% and severe depression symptoms was 10%. As shown in (Table 3) 29% of participants with severe anxiety symptoms experienced progression delay and 30% of participants with severe depression symptoms experienced progression delay. Finally, 27% of participants who listed having no general support experienced progression delay. The bivariate analysis indicated a high correlation between depression (X^2^ (4, N = 1 642) = 22.79, *p* < 0.001), anxiety symptoms (X^2^ (3, N = 1 642) = 12.25, *p* = 0.007) and progression.

**Table 3:** Summary of mental health and social support variables of the sample

Variable	Successful Progression (n = 1 240)	Progression Delay (n = 402)	*p-value*
**Anxiety symptoms severity**			0.007
Minimal	383 (80%)	93 (20%)	
Mild	398 (76%)	126 (24%)	
Moderate	246 (72%)	94 (28%)	
Severe	213 (71%)	89 (29%)	
**Depression symptoms severity**			< 0.001
Minimal	241 (83%)	49 (17%)	
Mild	430 (78%)	118 (22%)	
Moderate	300 (72%)	114 (28%)	
Moderate severe	161 (69%)	74 (31%)	
Severe	108 (70%)	47 (30%)	
**Main source of general support**			0.056
Both parents	619 (78%)	171 (22%)	
Single parents	350 (74%)	124 (26%)	
Grandparent(s) or guardian(s)	146 (69%)	65 (31%)	
Other family and/or friends(s)	58 (74%)	20 (26%)	
Spouse/ partner	11 (92%)	1 (8%)	
No support	56 (73%)	21 (27%)	

The multivariate logistic regression (Table 4) showed that being enrolled in the Engineering field of study increased the likelihood of progression delay more than nine-fold (adjusted odds ratio (aOR) = 9.33, 95% CI: 6.35–13.72, *p* < 0.001) and of Science more than four-fold (aOR = 4.23, 95% CI: 2.88–6.22, *p* < 0.001). Furthermore, experiencing moderate depression symptoms increased the adjusted odds of progression delay almost two-fold (aOR = 1.98, 95% CI: 1.30-3.00, *p* = 0.001), while moderate severe symptoms of depression increased the likelihood of progression delay almost three-fold (aOR = 2.70, 95% CI:1.70–4.30, *p* < 0.001). Severe depression symptoms also increased the odds of progression delay almost three-fold (aOR = 2.59, 95% CI:1.54–4.35, *p* < 0.001). An increase in the severity of depression symptoms was also found to lead to a higher likelihood of progression delay. Anxiety symptoms did not meet the threshold to be included in the final model.

**Table 4:** Logistic regression model used to calculate the predictors of academic performance

Variable	aOR (95% CI)	*p*-value	Standard error
**Sex**			
Female	ref		
Male	1.10 (0.84–1.45)	0.464	0.152
**Field of Study**			
Humanities	ref		
Engineering	9.33 (6.35–13.72)	< 0.001	1.834
Health Sciences	1.56 (0.99–2.52)	0.057	0.378
Commerce, Law and Management	1.17 (0.71–1.90)	0.541	0.291
Science	4.23 (2.88–6.22)	< 0.001	0.831
**Race** ^**a**^			
Black African	ref		
Coloured	0.93 (0.42–2.06)	0.867	0.377
Indian	0.96 (0.62–1.47)	0.846	0.211
White	0.62 (0.37–1.03)	0.065	0.161
**Major depression disorder symptom severity**			
Minimal	ref		
Mild	1.40 (0.93–2.11)	0.103	0.291
Moderate	1.98 (1.30-3.00)	0.001	0.421
Moderate severe	2.70 (1.70–4.30)	< 0.001	0.639
Severe	2.59 (1.54–4.36)	< 0.001	0.688
**High school quintile**			
1	ref		
2	0.87 (0.49–1.53)	0.621	0.250
3	1.11 (0.66–1.87)	0.695	0.296
4	0.83 (0.49–1.50)	0.579	0.244
5	0.50 (0.30–0.85)	0.010	0.135
Other	0.47 (0.26–0.83)	0.009	0.136
**NSFAS financial aid support recipient**			
No	ref		
Yes	0.67 (0.26–0.83)	0.007	0.100

^a^ Chinese and unknown race categories were dropped due to low number and/or zero exposure in the control population. This table provides adjusted Odds Ratios (aOR) and 95% CI formatted as “OR (lower CI-upper CI)” for each result of logistic regression

Two variables, high school quintile (quintile 5 and other) and receiving financial aid from the National Student Financial Aid Scheme (NSFAS), decreased the odds of progression delay. Participants who completed Grade 12 in well-resourced high schools (high school quintile 5), and those whose high school was classified as ‘Other’ (private and international high schools) were also significantly less likely to experience progression delay (aOR = 0.50, 95% CI:0.30–0.85, p = 0.01) and (aOR = 0.47, 95% CI:0.26–0.83, p = 0.009), respectively. Participants who received financial aid support from the NSFAS were also significantly less likely to experience progression delay (aOR = 0.67, 95% CI:0.26–0.83, p = 0.007).

## Discussion

The prevalence of severe anxiety symptoms was 18% and severe depression symptoms was 10%, when using standardized tools. These findings on anxiety and depression corroborate a recent South African study that found a 21% prevalence of generalized anxiety disorder and 14% prevalence of major depressive disorder among first-year university students when using a 12-month recall [3]. Findings from international literature vary with studies reporting depression and anxiety levels as high as 54% and 66%, respectively [6]. It is important to stress that the current study presents findings for anxiety and depression symptoms and not major depressive disorder or generalised anxiety disorder.

Findings from our study suggest that depression symptoms are predictive of progression delay, confirming the hypothesis underpinning this study. In fact, results indicate that moderate depression symptoms increased the odds of progression delay almost two-fold and that moderate severe and severe depression symptoms increased the adjusted odds of progression delay by three-fold. These findings align with both South African literature, which found that students experiencing major depressive disorder were almost four times more likely to perform poorly [3] and international literature from the USA and the UAE which associated low GPA scores with depression [18, 19]. Anxiety symptoms were not significant in their association with progression delay in the logistic regression, also a common finding [19, 30, 31].

Common mental disorders may affect academic performance in a number of ways. One way is class attendance, which is an important contributor to academic success [30]. Evidence from universities in Australia and Jordan found that students experiencing common mental disorders, including depression, on average had higher levels of class absenteeism compared to students not experiencing mental disorders [20, 32]. In their work, Eisenberg et al., (2009) conceptualize the impact of poor mental health on academic performance. In it they emphasize the potential impact of mental illness, including depression, in the acquisition of cognitive skills [2]. Depression symptoms, such as having low energy and difficulty concentrating [2, 7, 30], impact on non-cognitive skills that include persistence and motivation, which have a direct effect on cognitive function and thus the acquirement of knowledge. The presence of depression impacts on these non-cognitive skills resulting in low academic productivity, leading to potentially lower skill acquisition as reflected by lower scores [2].

It is also important to note the potential bi-directionality of the above trend. It is plausible that academic failure, including progression delay, may increase the risk of depression symptoms [2]. Other literature investigating depression, academic achievement and absenteeism, has acknowledged this [32]. Furthermore, findings from Nigeria, for instance, report that students experiencing academic failure often report feelings of anger, shame, disappointment and hopelessness [33, 34]. Research has also highlighted the compounding effects of academic failure, including the additional financial stress of having to re-register and also the time commitment due to increased workloads, all which can have significant implications on mental health [34].

The current research also found that 24% of the first-time, first-year undergraduate university students experienced progression delay during a single year at a large South African tertiary institution. These levels are consistent with findings from a similar study which found academic failure to be 26% among first-year South African university students in the Western Cape province [3]. These findings are difficult to compare with international literature that typically measures academic performance using GPA. In terms of student success, variables such as sex, race, high school quintile and field of study have been well documented as predictors of academic success [13, 17, 35] and in the current research, these were also found to significantly impact academic performance. The results of our study on sex and race are supported by other research findings, both in South Africa and internationally, which indicate that female students often outperform their male counterparts [3, 30], and that White students often attain the highest academic scores [13, 30].

The results indicated that financial aid (NSFAS) as well as attending well-resourced high schools (quintile 5 and other) protected against progression delay. These findings are in line with literature that has demonstrated that students from well-resourced schools perform better academically than students from poorer schools [17]. Furthermore, studies have found that students with a financial need who receive financial aid were more likely to be academically successful when compared to their counterparts without any state funding [15, 36].

Students registered in the field of study of Engineering had a probability of progression delay nine times higher than Humanities where 89% of students successfully progressed, while students registered in the Science field had an increased likelihood of progression delay by a factor of four. These findings correspond with previous research that suggests that students enrolled in science, technology, engineering and mathematics (STEM) fields, across institutions, grapple with the curriculum [37]. In fact, although enrolment in the STEM field have increased over time in South Africa, they account for the lowest university success and graduation rates [38].

Our findings highlight the important relationship between student mental health and academic progression, suggesting that student mental health should be recognized as a critical component of academic performance at universities. As such, universities should consider making provisions for mental wellness resources on campus, and build institutional cultures that promote mental wellness. However, mental health is a public health issue, and it is unreasonable to expect universities to be the sole drivers of change. We believe that the student voice is essential to reconciling the roles of both universities and the health care system in improving student academic performance.

## Conclusion

We present original findings from a study involving first-time, first-year undergraduate students at a large South African university. Although the generalizability of the findings may be limited, our data builds on a growing body of literature demonstrating the negative impact that depression symptoms have on student academic performance, through delayed student progression and ultimately potentially low student throughput rates. It is important for students, universities as well as government departments to recognize the impact of mental health on student performance and work together to identify student’s mental health needs and how these can be met. Holistic student support programmes offered by universities should work towards fully incorporating student mental wellness activities. The current study has a number of strengths, including: a large study population; being carried out at an institution with a diverse student population; and experiencing a high response rate (32%) when compared to similar studies. To our knowledge, this is one of the largest studies, in terms of the sample size and response rate, on the African continent to explore the relationship between depression symptoms and academic performance. Studies using similar methodological approaches typically achieve response rates between 8 and 13% [3, 22, 23].

However, a number of limitations should also be considered when interpretating the results. The PHQ-9 is used as a screening tool for depression symptoms and is not a diagnostic clinical tool. The PHQ-9 has, however, been validated and determined to give accurate accounts of the prevalence of depression [25]. The current study was carried out at a single university, with differences delineated between study participants and non-participants, which limits the generalizability of the current findings. Furthermore, bias could have been introduced due to self-selected sampling. Finally, the cross-sectional nature of the study limits establishment of causality.

The students who participated in the current study have subsequently been invited to participate in similar research during the initial COVID-19 pandemic years (2020–2021) and the first “post-COVID-19 year” (2022). This follow-up study will help us understand the impact of the COVID-19 pandemic on the mental health of these South African students.

## Electronic supplementary material

Below is the link to the electronic supplementary material.

Supplementary Material 1

## Data Availability

The data that support the findings of this study are available from the University Registrar of the University of the Witwatersrand, Johannesburg, but restrictions apply to the availability of these data, which were used under license for the current study, and so are not publicly available. Data are however available from the corresponding author upon reasonable request and with permission of the University Registrar of the University of the Witwatersrand, Johannesburg.
