# Supplementary material for: The relationship between depression symptoms and academic performance among first-year undergraduate students at a South African university: a cross-sectional study
Source: BMC Public Health. 2022 Nov 11;22:2067. doi: 10.1186/s12889-022-14517-7 (PMC9651123; doi:10.1186/s12889-022-14517-7)
Supplement: Supplementary file 1 — Supplementary Material 1 [file 12889_2022_14517_MOESM1_ESM.docx]

**Supplementary tables**

**Chi-square test of independence for progression status, anxiety symptoms and sources of general support health variables and depression symptoms**

| **Variable** | Depression Severity | | | | | |
| --- | --- | --- | --- | --- | --- | --- |
|  | Minimal  (n= 290) | Mild  (n= 548) | Moderate  (n=414) | Moderate Severe  (n=235) | Severe  (n=155) | *p-value* |
| **Progression status** |  |  |  |  |  | <0.001 |
| Progression | 241 (19%) | 430 (35%) | 300 (24%) | 161 (13%) | 108 (9%) |  |
| Progression delay | 49 (12%) | 118 (30%) | 114 (28%) | 74 (18%) | 47 (12%) |  |
| **Anxiety symptoms severity** |  |  |  |  |  | <0.001 |
| Minimal | 238 (50%) | 189 (40%) | 38 (8%) | 9 (2%) | 2 (0%) |  |
| Mild | 48 (9%) | 272 (52%) | 163 (31%) | 36 (7%) | 5 (1%) |  |
| Moderate | 3 (1%) | 70 (21%) | 147 (43%) | 91 (27%) | 29 (8%) |  |
| Severe | 1 (0%) | 17 (6%) | 66 (22%) | 99 (33%) | 119 (39%) |  |
| **Main source of general support** |  |  |  |  |  | 0.005 |
| Both parents | 160 (20%) | 270 (34%) | 194 (25%) | 107 (14%) | 59 (7%) |  |
| Single parents | 73 (15%) | 158 (33%) | 124 (26%) | 69 (15%) | 50 (11%) |  |
| Grandparent(s) or guardian(s) | 31 (16%) | 66 (35%) | 51 (27%) | 30 (16%) | 12 (6%) |  |
| Other family and/or friends(s) | 8 (10%) | 31 (40%) | 18 (23%) | 12 (15%) | 9 (12%) |  |
| Spouse/ partner | 1 (8%) | 4 (33%) | 4 (33%) | 1 (8%) | 2 (17%) |  |
| No support | 14 (18%) | 16 (21%) | 16 (21%) | 13 (17%) | 18 (23%) |  |
